# Post-transplant anemia and associated risk factors: the impact of steroid-free therapy

**DOI:** 10.1590/1516-3180.2013.1316523

**Published:** 2013-12-01

**Authors:** Claudia Maria Costa Oliveira, Paula Sátiro Timbó, Sanna Roque Pinheiro, Janaína Gonçalves Silva Leite, Luciana Sátiro Timbó, Ronaldo Matos Esmeraldo

**Affiliations:** I MD, PhD. Nephrologist in the Transplantation Department, Hospital Geral de Fortaleza, and Associate Professor, Discipline of Nephrology, Faculdade de Medicina Christus, Fortaleza, Ceará, Brazil; II Medical Student in the Nephrology Department, Faculdade de Medicina Christus, Fortaleza, Ceará, Brazil; III MD. Director of the Transplantation Department, Hospital Geral de Fortaleza, Fortaleza, Ceará, Brazil

**Keywords:** Anemia, Kidney transplantation, Steroids, Risk, Therapy [subheading], Anemia, Transplante de rim, Esteróides, Risco, Terapia

## Abstract

**CONTEXT AND OBJECTIVE:**

The prevalence of post-renal transplant anemia (PTA) reported in the literature is variable and several factors contribute towards its pathophysiology. This study aimed to investigate the prevalence of PTA, its associated risk factors and the impact of therapy without steroids.

**DESIGN AND SETTING:**

Retrospective cohort study in a renal transplantation unit at a tertiary hospital.

**METHODS:**

Anemia was defined as hemoglobin (Hb) < 12 g/dl in female adult recipients and < 13 g/dl in males. Donor and recipient age and gender, type of donor, creatinine, delayed graft function, acute rejection, use of angiotensin-converting enzyme inhibitors (ACEI) or angiotensin receptor blockers (ARB) and therapy without steroids were investigated as risk factors for PTA through multivariate logistical regression analysis.

**RESULTS:**

Evaluations were performed on 258 recipients (mean age: 38.8 years; 60.5% males; 35.7% did not receive steroids). Anemia was diagnosed in 38% of the patients (at the sixth month, M6), 28% (M12), 32% (M24) and 45% (at last follow up). Donor age > 50 years was associated with greater risks of PTA at M6 (odds ratio (OR) = 4.68) and M24 (OR = 6.57), as well as with therapy without steroids at M6 (OR = 2.96). Delayed graft function was independently associated with PTA at M6 (OR = 3.66) and M12 (OR = 2.85).

**CONCLUSION:**

The lowest prevalence of PTA was observed between M9 and M24 after renal transplantation. Delayed graft function, donor age and therapy without steroids were the most important factors associated with PTA.

## Abstract (pt)

**CONTEXTO E OBJETIVO:**

A prevalência de anemia pós-transplante renal (APR) relatada na literatura é variável e vários fatores contribuem para sua fisiopatologia. Este estudo objetivou investigar a prevalência de APR, os fatores de risco associados e o impacto da terapia sem esteroides.

**TIPO DE ESTUDO E LOCAL:**

Estudo de coorte retrospectivo em unidade de transplante renal em hospital terciário.

**MÉTODOS:**

Anemia foi definida como hemoglobina (Hb) < 12 g/dl em receptores adultos do sexo feminino e < 13 g/dl no masculino. Idade e gênero do doador e do receptor, tipo de doador, creatinina, função retardada do enxerto (FRE), rejeição aguda, uso de inibidores da enzima conversora da angiotensina (IECA)/bloqueadores dos receptores da angiotensina (BRA) e terapia sem esteroides foram investigados como fatores de risco para APR em análise de regressão logística multivariada.

**RESULTADOS:**

Duzentos e cinquenta e oito receptores foram avaliados (idade média: 38,8 anos; 60,5% homens; 35,7% em terapia sem esteroides). Anemia foi diagnosticada em 38% no sexto mês (M6), 28% (M12), 32% (M24) e em 45% dos pacientes na última data de acompanhamento. Idade do doador > 50 anos associou-se a maior risco de APR aos 6 (*odds ratio*, OR = 4,68) e 24 meses (OR = 6,57), bem como terapia sem esteroides aos 6 meses (OR = 2,96). FRE associou-se independentemente com APR aos 6 (OR = 3,66) e 12 meses (OR = 2,85).

**CONCLUSÃO:**

A menor prevalência de APR foi observada entre 9 e 24 meses pós-transplante renal. FRE, idade do doador e terapia sem esteroides foram os principais fatores associados à APR.

## INTRODUCTION

The prevalence of post-renal transplant anemia reported in the literature is variable (13-70%)^1^ ^,^ ^2^ and its management remains poorly explored. The variable prevalence of post-renal transplant anemia depends on the definition of anemia that is applied, the characteristics of the population studied and the length of time after transplantation.

Post-renal transplant anemia may affect quality of life and cardiovascular morbidity/mortality. It contributes towards left ventricular hypertrophy and heart failure, thus increasing the risk of cardiovascular events, which are considered to be the main causes of death after transplantation.^3^ ^,^ ^4^

Several factors such as graft function, use of azathioprine, mycophenolate mofetil or angiotensin inhibitors, the donor's age, viral infections (cytomegalovirus and parvovirus B19), inflammation, hemolytic anemia, hemolytic-uremic syndrome and acute rejection^1^ ^,^ ^5^ ^,^ ^6^ contribute towards the pathophysiology of post-renal transplant anemia.

## OBJECTIVE

This study was designed to evaluate the prevalence of anemia at different times after transplantation; to investigate the associated risk factors; and to evaluate the association between anemia and therapy with or without steroids. 

## METHODS

We conducted a retrospective cohort study on patients who underwent kidney transplantation in a single hospital between January 2001 and May 2006. The study included all patients who received a transplant during the period of the study and who were older than 18 years, with at least two years of post-transplant follow-up. This study was approved by the Research Ethics Committee of Hospital Geral de Fortaleza, and data were obtained using a post-transplant follow-up form. Pregnant patients were excluded. Data were obtained from the patient's medical files. Over the period of the study, 378 transplantations were performed, and 120 recipients were not included because they did not meet the inclusion criteria.

Anemia was defined as hemoglobin (Hb) < 12 g/dl in women and < 13 g/dl in men (World Health Organization and American Society of Transplantation criteria7). Post-renal transplant anemia was classified as moderate when Hb > 11 and < 12 g/dl (male) and > 10 and ≤ 11 g/dl (female), and severe when Hb ≤ 11 g/dl (male) and ≤ 10 g/dl (female). The prevalence of post-renal transplant anemia was investigated at 1, 2, 3, 4, 5, 6, 9, 12 and 24 months after transplantation. 

Donor and recipient age and gender, type of donor, creatinine, delayed graft function (DGF), acute rejection and therapy without steroids were investigated as risk factors for post-renal transplant anemia at 6, 12 and 24 months after transplantation. 

Data were expressed as the mean ± standard deviation (SD). The correlation between Hb and creatinine was assessed through Pearson's correlation coefficient. P values < 0.05 were considered statistically significant. We first determined the parameters that were associated with post-renal transplant anemia at 6, 12 and 24 months using univariate analysis between the groups by means of a two-tailed independent-sample Student's t test or Fisher's test with significance taken at P = 0.10. The variables that reached a P value < 0.10 in the univariate analysis were included in a multivariate, stepwise logistic regression analysis to investigate the risk factors associated with post-renal transplant anemia at 6, 12 and 24 months after transplantation in the study population. All odds ratios (ORs) were reported with a 95% confidence interval. The Statistical Package for Social Sciences (SPSS) 14.0 software was used for statistical analysis.

## RESULTS

The population studied comprised 258 renal transplant recipients with a mean age of 38.8 ± 11.4 years, and 60.5% of the sample were male. The primary renal disease was unknown in 38% of the cases; glomerulonephritis was diagnosed in 25% of cases; and hypertension was diagnosed in 19%. A total of 53.5% of the patients received a graft from a deceased donor. The initial immunosuppressive therapy consisted of mycophenolate mofetil in 92.2% of the patients, cyclosporine in 45%, tacrolimus in 53% and rapamycin in 2.3%. Prednisone was not used in maintaining the immunosuppressive therapy in 35.7% of the patients. Induction therapy with basiliximab was administered to 153 patients (59.3%), and thymoglobulin was administered to 11 patients (4.3%). Delayed graft function, defined as the need for dialysis in the first week post-transplant, and clinical or biopsy-proven acute rejection were observed in 38% and 14.7% of the recipients, respectively. The demographic characteristics of the population studied and the mean hemoglobin and creatinine values over the course of the follow-up are presented in Table 1.

**Table 1:** Demographic and laboratory data of the study population

Number of patients	258
Recipient gender	Male: 156 (60.5%)
Recipient age	38.8 ± 11.4 years
Donor gender	Male: 149 (57.8%)
Donor age	31.6 ± 11.3 years
Type of donor	Cadaveric: 53.5%
Follow-up	5.4 ± 1.9 years
Acute rejection	14.7%
Delayed graft function	38%
Use of ACEI/ARB	55.4%
Cyclosporine	45%
Tacrolimus	53%
Mycophenolate mofetil	92.2%
Prednisone	74.3%
Rapamycin	2.3%
Hemoglobin at 1 months	11.07 ± 1.83 g/dl
Hemoglobin at 2 months	11.93 ± 2.03 g/dl
Hemoglobin at 3 months	12.24 ± 2.11 g/dl
Hemoglobin at 6 months	13.19 ± 2.12 g/dl
Hemoglobin at 12 months	13.66 ± 2.03 g/dl
Hemoglobin at 24 months	13.50 ± 2.11 g/dl
Hemoglobin at the last follow-up	12.56 ± 2.01 g/dl
Creatinine at discharge	2.29 ± 1.85 mg/dl
Creatinine at 1 month	1.93 ± 1.59 mg/dl
Creatinine at 2 months	1.51 ± 0.70 mg/dl
Creatinine at 3 months	1.39 ± 0.57 mg/dl
Creatinine at 6 months	1.41 ± 0.62 mg/dl
Creatinine at 12 months	1.29 ± 0.76 mg/dl
Creatinine at 24 months	1.32 ± 0.66 mg/dl
Creatinine at the last follow-up	1.63 ± 1.50 mg/dl

ACEI/ARB = angiotensin-converting enzyme inhibitor/angiotensin receptor blockers

The average hemoglobin level at the time of transplantation was 11.8 g/dl, and 63.2% of patients were classified as having anemia at the time of the surgery. The mean hemoglobin increased from 11.7 g/dl in the first month after transplantation to 13.6 g/dl at 12 months.

**Figure 1:**
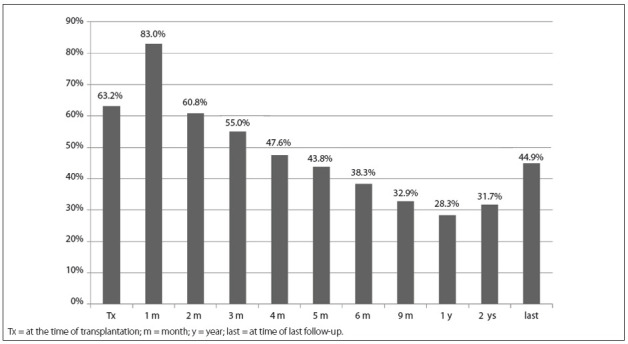
Prevalence of post-renal transplant anemia in the study population at different times after transplantation.

Anemia was diagnosed in 83% of the patients at one month, 60.8% at two months, 55% at three months, 47.6% at four months, 43.8% at five months, 38.3% at six months, 32.9% at nine months, 28.3% at one year, 31.7% at two years and 44.9% at the last follow-up (mean time after transplantation: 5.4 ± 1.9 years) (Figure 1). The anemia was classified as moderate in 11.6%, 9.3% and 10.1% of the patients at 6, 12 and 24 months, respectively, and severe in 11.0%, 5.0% and 5.8% at the same time points (Table 2).

**Table 2:** Classification of post-renal transplant anemia in the study group

Anemia	Moderate	Severe
6 months	11.6%	11.0%
12 months	9.3%	5.0%
24 months	10.1%	5.8%

A significant negative correlation was found between serum creatinine and hemoglobin levels at all of the time points after transplantation (Table 3).

**Table 3:** Correlation between creatinine and hemoglobin in the study group

Hemoglobin/creatinine	Pearson's correlation r	P
6 months	-0.137	0.029
12 months	-0.175	0.005
24 months	-0.232	< 0.0001
Last follow-up	-0.419	< 0.0001

The factors that were significantly associated with post-renal transplant anemia at six months after transplantation were donor age, DGF, creatinine at discharge, type of donor and therapy without steroids (Table 4). The predictive factors for post-renal transplant anemia at 12 months were donor age, DGF, use of angiotensin-converting enzyme inhibitor (ACEI) or angiotensin receptor blockers (ARB) and creatinine at 12 months. The predictive factors for post-renal transplant anemia at 24 months were donor age, DGF, type of donor, therapy without steroids, use of ACEI/ARB and creatinine at 12 and 24 months.

**Table 4:** Factors associated with post-renal transplant anemia at 6, 12 and 24 months (m), expressed by means of multivariate analysis

Variable	Follow-up	OR [95% CI]	P
Donor age > 50 years	6 m	4.68 [1.3-16.9]	0.018
24 m	6.57 [1.67-25.81]	0.007
Therapy without steroids	6 m	2.96 [1.65-5.28]	< 0.0001
Delayed graft function	6 m	3.66 [1.69-7.88]	0.001
	12 m	2.85 [1.45-5.57]	0.002

OR = odds ratio

CI = confidence interval.

Donor age > 50 years was independently associated with increased risk of post-renal transplant anemia at six months (OR = 4.68 [1.3-16.9]; P = 0.018) and 24 months after transplantation (OR = 6.57 [1.67-25.81]; P = 0.007), as was therapy without steroids at six months (OR = 2.96 [1.65-5.28]; P < 0.0001). Delayed graft function was independently associated with the risk of post-renal transplant anemia at six months (OR = 3.66 [1.69-7.88]; P = 0.001) and 12 months after transplantation (OR = 2.85 [1:45 to 5:57]; P = 0.002) (Table 4).

## DISCUSSION

After kidney transplantation, many factors that contribute towards anemia in chronic kidney disease (CKD) persist, but causes specific to transplantation may predominate. During the immediate post-transplant period, the main causes of anemia are CKD residual anemia, blood loss during surgery, DGF and acute rejection. Later on, chronic rejection with graft dysfunction and immunosuppression may result in recurrence of anemia.^8^

Post-renal transplant anemia may contribute towards damage to and subsequent loss of the graft and progression of CKD.^9^ ^,^ ^10^ Tissue hypoxia stimulates synthesis of transcription factors that promote fibrosis and progression of chronic graft nephropathy.^11^ Some authors have suggested that post-renal transplant anemia may contribute towards graft loss and evolution of chronic nephropathy, with a 38% increased risk of graft loss.^12^ Moreover, correction of anemia using erythropoietin, which has anti-apoptotic effects on erythrocyte precursor cells in bone marrow, due to inhibition of the caspase pathways,^13^ is speculatively thought to protect against the destruction of tubular cells due to apoptosis, as demonstrated in other animal tissues. These factors emphasize the importance of post-renal transplant anemia and the need for studies that assess it.

The population studied here included young patients (mean age: 39 years). The cohort was 60% male and 53.5% received their transplant from a deceased donor. According to the Brazilian Registry of Organ Transplants,14 out of the 25,434 kidney transplantations made over a 10-year period in Brazil, 44.3% were from a deceased donor. In an European multicenter study that evaluated post-renal transplant anemia in a larger number of patients, 62% of the patients were male, the mean age was 48 years and 89.7% of the transplants were from a deceased donor.^1^ Therefore, our population included younger patients and had a lower prevalence of deceased donors. However, the European study evaluated post-renal transplant anemia in patients for different periods of time after the transplantation (six months and one, three and five years), and the overall prevalence of anemia at the time of enrollment in the study was 38.6%.^1^

### Prevalence of post-renal transplant anemia

Despite being a well-known pathological condition, post-renal transplant anemia has not been thoroughly studied. Furthermore, there are no Brazilian studies on the prevalence of post-renal transplant anemia that have evaluated the risk factors associated with its development. Previous studies have shown that post-renal transplant anemia is more prevalent in children, African-Americans and diabetics.^15^ ^,^ ^16^ In addition, anemia is more prevalent in kidney transplant recipients than in patients with chronic kidney disease of the native kidneys with the same level of renal function.^17^

The prevalence of post-renal transplant anemia described in the literature has ranged from 13 to 70% irrespective of the time elapsed after transplantation. A number of studies reporting on varying numbers of patients have been conducted.^1^ ^,^ ^12^ ^,^ ^16^ ^,^ ^18^ ^-^ ^24^ This varying prevalence of PTA probably is observed because different criteria have been suggested for the definition of anemia, including Hb < 13 g/dl for male patients and < 12 g/dl for females;^16^ Hb < 13.5 g/dl for males and < 12 g/dl for females (K/DOQI);^25^ Hb < 12 g/dl for males and < 11 g/dl for females (European Best Practice Guidelines);^26^ Hb < 12.5 for males and < 11.5 g/dl for females;^18^ Hb < 11 after three months;^19^ and hematocrit (Hct) < 33%.^8^ ^,^ ^16^ ^,^ ^21^

The prevalence of post-renal transplant anemia in the present study was 38.3% at six months and 28.3% at 12 months, and these results are similar to those reported by Turkowski et al., who reported that 35.5% of patients had anemia at six months, and 25% at 12 months after transplantation.^27^ However, Shibagakin et al. analyzed 192 patients and found that 41.1% had anemia at six months, and 44.8% at 12 months after transplantation.^15^

The prevalence of post-renal transplant anemia follows a bimodal pattern with an initial peak at the time of transplantation, when erythropoiesis is minimal due to various factors, and another peak after the third year, which is associated with the progression of renal disease. Normal hemoglobin levels are expected as early as two months after transplantation in patients without iron deficiency and without graft dysfunction.^28^

In our study, two months after transplantation, we observed high prevalence of anemia (60.8%), which was similar to what was detected at the time of transplantation (63.2%). We found a progressive decrease in the prevalence of anemia up to 12 months (28.3%) and a subsequent progressive increase, observed at two years (31.7%) and five years (44.9%). Yorgin et al. evaluated 128 patients and found that the prevalence of anemia (Hct < 33%) was 43% at the time of transplant, 12% at one year, and 26% at five years.^2^ On the other hand, according to Mix et al., the prevalence of anemia (Hct < 36%) at the time of transplant was 76%, and it reduced to 12% at one year.^29^

Restoration of endogenous secretion of erythropoietin (EPO) and its impact on the outcome of anemia have been assessed in several studies. After an initial peak in EPO levels during the first days post-transplant, restoration of EPO synthesis depends on the recovery of renal function.^28^

### Predictive factors for post-renal transplant anemia

*Creatinine*

In this study, creatinine > 2 mg/dl was not predictive of post-renal transplant anemia at any of the time points studied.

EPO synthesis decreases and resistance to its effects increases when renal function deteriorates.^30^ Therefore, an inverse correlation between function and hemoglobin is expected. Many studies have found renal function to be a predictor of post-renal transplant anemia, although this was not the case in the present study. In a multicenter European study,^1^ creatinine > 2 mg/dl was a risk factor for post-renal transplant anemia. In an article by Yorgin et al., urea nitrogen and creatinine were risk factors for post-renal transplant anemia at 12 months after transplantation.^16^ Other authors have found platelets (on day 7 post-transplant), presence of DGF, creatinine clearance, creatinine levels at 12 months and Hb at six months to be predictors of post-renal transplant anemia after 12 months.^25^ Unal et al. found that creatinine clearance was an independent risk factor for post-renal transplant anemia in a multivariate analysis,^22^ and Shah et al. reported that glomerular filtration rate (GFR) was the most important predictor of post-renal transplant anemia.^23^ More recently, Molnar et al.^31^ and Choukroun et al.^32^ also found that hemoglobin concentration was significantly correlated with the estimated glomerular filtration rate (eGFR).

The degree of anemia is about 10 times higher for a given degree of renal dysfunction when recipients of a kidney transplant are compared with native kidney disease patients.^17^

The choice of creatinine as a marker of renal function rather than creatinine clearance may have been a limitation of the present study. Creatinine is an insensitive and late marker of renal dysfunction and glomerular filtration rate must be preferred to estimate renal function,^33^ which may have been responsible for the results.

*Donor age*

In the present study, donor age > 50 years was associated with presence of post-renal transplant anemia (OR = 4.68 at six months and OR = 6.57 at 24 months). In the European study, donor age > 60 years was a risk factor for post-renal transplant anemia (OR = 1.41).^1^ Since donor age was an independent risk factor for post-renal transplant anemia, the association could not be explained by the lower degree of renal function associated with kidneys from older donors.

*Delayed graft function*

In the pre and immediate post-transplant period, ischemia-reperfusion injury and calcineurin inhibitor nephrotoxicity may lead to tubular cell death and alloimmune fibrosis, with consequent DGF, acute cell rejection, tissue fibrosis, poor graft function and graft loss.^34^ DGF has a negative impact on long term graft function, since it increases patient morbidity and mortality.^35^ DGF is associated with increased biopsy-proven acute rejection as a consequence of increased immunogenicity of the ischemic graft.^34^ Few studies have included DGF in the investigation of post-renal transplant anemia predictors.

Chhabra et al. showed that the prevalence of DGF was higher in anemic patients (Hb < 11 g/dl) than in those with Hb > 11 g/dl (32% versus 23%; P = 0.027).^19^ The European study did not include DGF in the analysis of post-renal transplant anemia. In the present study, DGF was a predictor of post-renal transplant anemia at six and 12 months after transplantation, independently of acute rejection and renal function. The prevalence of DGF (38%) was similar to what was found in a recent European multicenter study, which evaluated whether the use of high doses of EPO immediately after transplantation would have an impact on the prevalence of DGF.^36^

*Immunosuppression without steroids*

Recently, studies have been conducted on the safety, efficacy and toxicity of kidney transplantation without the use of steroids, and the number of studies in this area has been increasing.^37^ ^-^ ^40^

Steroid-free therapy for kidney transplant patients has been recognized to be beneficial because of the complications associated with long-term steroid use, such as weight gain, osteopenia, hypertension, hyperglycemia and increased risk of infection.^41^

In clinical practice, it has been observed that leukocyte levels in the absence of steroid use are lower than in patients who use steroids. The prevalence of anemia with or without steroid use has been studied and deserves further evaluation. In the present study, steroid-free therapy was found to be a risk factor for post-renal transplant anemia six months after transplantation, thus suggesting that steroids affect erythropoiesis.

The role of glucocorticoids in erythropoiesis is unclear. Steroids have been used to treat aplastic anemia and can improve the production of red blood cells in cases of congenitally abnormal erythropoiesis (Diamond-Blackfan syndrome). Golde et al. reported that dexamethasone boosts the proliferation of red blood cell progenitors stimulated by erythropoietin.^42^ The effect of dexamethasone may involve increased sensitivity of progenitor cells to erythropoietin in vitro.

It has been shown that steroids increase the growth of colonies in vitro in normal bone marrow,^43^ ^,^ ^44^ and that treatment of non-anemic patients with prednisone appears to increase erythropoiesis.^45^ According to Von Lindern et al., the glucocorticoid receptor cooperates with the activated erythropoietin receptor and with the receptor for stem cell factor (SCF), to enhance and sustain proliferation of erythroid progenitors in vitro.^46^

In the study by Chhabra et al., a greater proportion of patients in the anemic group were on prednisone than in the non-anemic group.^19^ This was the opposite of what we found in our study. However, those authors did not justify their finding. Previous studies observed increased anemia in the steroid-free group early after transplantation, but this usually normalizes later on in the post-transplant period, probably as a result of increased ESA use. Guitard et al. observed that a daily dosage of steroids < 0.3 mg/kg at one month after transplantation was strongly associated with anemia at six and 12 months after orthotopic liver transplantation.^47^

More recently, Jones et al found that prednisone was independently predictive of decreased hemoglobin in a cohort of 530 kidney allograft recipients, but they did not discuss or justify this observation.^48^

*Angiotensin-converting enzyme inhibitor (ACEI) and angiotensin receptor blockers (ARB)*

Other drugs that have been associated with post-renal transplant anemia include inhibitors of the renin-angiotensin-aldosterone system (RAAS). RAAS is an important regulator of erythropoiesis, and its inhibition has been shown both to be associated49 and not to be associated^50^ with post-renal transplant anemia. Use of angiotensin-converting enzyme inhibitor/angiotensin receptor blockers (ACEI/ARB) was predictive of post-renal transplant anemia in the European study,^1^ but not in the study by Chhabra et al.^19^

Transplant patients are more susceptible to RAAS inhibition, which may indicate that there is a greater degree of activation of RAAS in these patients. In the present study, RAAS blockade was predictive of post-renal transplant anemia at 12 and 24 months according to the univariate analysis. Although 55.4% of patients were treated with RAAS inhibitors in our study, the number of patients was insufficient for this to remain a factor in the multivariate analysis.

*Other variables studied*

In our study, the prevalence of acute rejection was low, which may have contributed towards the lack of association with the presence of anemia. The European multicenter study did not find acute rejection to be a risk factor for post-renal transplant anemia, although patients who experienced more episodes of treated acute rejection had lower mean Hb levels.^1^ The increased inflammatory response that accompanies rejection seems to downregulate genes that are involved in erythropoiesis,^51^ and it has been shown that occurrence of acute rejection in the first month after transplantation interrupts the erythropoietic response.^52^ Acute rejection is associated with lower Hb levels, but it is not an independent predictor of post-renal transplant anemia, since it is generally associated with worse renal function, use of higher doses of myelotoxic immunosuppressants to control new episodes of rejection and use of drugs to inhibit RAAS.

The immunosuppressants azathioprine and mycophenolate mofetil have been used in kidney transplant patients to prevent acute rejection. These drugs inhibit lymphocyte proliferation. They have myelotoxic side effects and are associated with the risks of developing anemia, leukopenia and thrombocytopenia. Furthermore, they have been clearly associated with the development of post-renal transplant anemia.^1^ It was not possible to evaluate this variable in the present study because 92.2% of the patients were taking mycophenolate mofetil.

## CONCLUSIONS

Post-renal transplant anemia had a high prevalence in our study, and the lowest prevalence of anemia was observed between nine months and two years after transplantation. The factors associated with post-renal transplant anemia were donor age > 50 years, therapy without steroids and DGF, in this population. Use of steroid-free therapy has been increasing over the last few years and the consequences of this therapy need to be better understood.

In the literature, there are few studies investigating steroids as predictors of post-renal transplant anemia and the results are divergent. Additional studies investigating the role of steroids in post-renal transplant anemia need to be conducted. To the best of our knowledge, the present study is the first to identify steroid-free therapy as a predictor of post-renal transplant anemia in kidney transplantation cases.
